# Mechanical Alterations Associated with Repeated Treadmill Sprinting under Heat Stress

**DOI:** 10.1371/journal.pone.0170679

**Published:** 2017-02-01

**Authors:** Olivier Girard, Franck Brocherie, Jean-Benoit Morin, Sébastien Racinais, Grégoire P. Millet, Julien D. Périard

**Affiliations:** 1 Aspetar Orthopaedic and Sports Medicine Hospital, Athlete Health and Performance Research Centre, Doha, Qatar; 2 ISSUL, Institute of Sports Sciences, University of Lausanne, Lausanne, Switzerland; 3 Laboratory Sport, Expertise and Performance (EA 7370), Research Department, French Institute of Sport (INSEP), Paris, France; 4 Université Côte d’Azur, LAMHESS, Nice, France; Norwegian University of Science and Technology, NORWAY

## Abstract

**Purpose:**

Examine the mechanical alterations associated with repeated treadmill sprinting performed in HOT (38°C) and CON (25°C) conditions.

**Methods:**

Eleven recreationally active males performed a 30-min warm-up followed by three sets of five 5-s sprints with 25-s recovery and 3-min between sets in each environment. Constant-velocity running for 1-min at 10 and 20 km.h^-1^ was also performed prior to and following sprinting.

**Results:**

Mean skin (37.2±0.7 *vs.* 32.7±0.8°C; P<0.001) and core (38.9±0.2 *vs.* 38.8±0.3°C; P<0.05) temperatures, together with thermal comfort (P<0.001) were higher following repeated sprinting in HOT *vs.* CON. Step frequency and vertical stiffness were lower (-2.6±1.6% and -5.5±5.5%; both P<0.001) and contact time (+3.2±2.4%; P<0.01) higher in HOT for the mean of sets 1–3 compared to CON. Running distance per sprint decreased from set 1 to 3 (-7.0±6.4%; P<0.001), with a tendency for shorter distance covered in HOT *vs.* CON (-2.7±3.4%; P = 0.06). Mean vertical (-2.6±5.5%; P<0.01), horizontal (-9.1±4.4%; P<0.001) and resultant ground reaction forces (-3.0±2.8%; P<0.01) along with vertical stiffness (-12.9±2.3%; P<0.001) and leg stiffness (-8.4±2.7%; P<0.01) decreased from set 1 to 3, independently of conditions. Propulsive power decreased from set 1 to 3 (-16.9±2.4%; P<0.001), with lower propulsive power values in set 2 (-6.6%; P<0.05) in HOT *vs.* CON. No changes in constant-velocity running patterns occurred between conditions, or from pre-to-post repeated-sprint exercise.

**Conclusions:**

Thermal strain alters step frequency and vertical stiffness during repeated sprinting; however without exacerbating mechanical alterations. The absence of changes in constant-velocity running patterns suggests a strong link between fatigue-induced velocity decrements during sprinting and mechanical alterations.

## Introduction

Accelerating over short distances is crucial in many team sports, where short-duration efforts (*e*.*g*., accelerated runs over 10–30 m or 3–5 s) are commonplace [1]. This is in turn associated with important moments in a game, such as gaining an advantage over an opponent or creating scoring opportunities. During football match play for instance, a straight sprint is most often observed prior to a goal being scored [2]. The ability to maintain sprint performance as maximal or ‘all out’ efforts are repeated (*i*.*e*., repeated-sprint ability) during a game is thereby crucial for athletes engaged in these disciplines.

An instrumented treadmill modified for sprint use, which allows athletes to run and freely dictate velocity (*i*.*e*., with no predetermined belt velocity imposed), is now available for the measurement of both valid [3,4] and reproducible [5] tri-dimensional ground reaction forces during each step of a sprint run. Continuous measurement of the kinetics/kinematics and calculation of spring-mass model characteristics have been performed using this treadmill to elucidate some of the biomechanical correlates of fatigue when sprinting repeatedly in temperate conditions [6–8]. These studies demonstrated an increase in contact time along with reductions in step frequency and vertical stiffness during a single series of repeated running sprints.

However, single-set repeated-sprint exercise (RSE) may not adequately reflect the complex match activity patterns observed in team sports. Consequently, the use of multiple-set RSE has recently been introduced in the repeated-sprint ability literature and shown to more accurately reflect the most intense phases of a game [8,9]. Utilizing this multiple-set RSE approach, Morin et al. [8] reported that all the mechanical variables reflecting force production and the effectiveness of force application progressively deteriorated across sets. That said, it is difficult to ascertain to which extent RSE-related alterations in running mechanics such as an increase in contact time, decrease in step frequency and vertical stiffness [6,8], result from the fatigue induced by running, or via changes in performance (*i*.*e*., mean running velocity and/or propulsive power). For example, in fresh or unfatigued conditions, increases and decreases in running velocity are accompanied by modifications in stride kinetics/kinematics and in spring-mass parameters [10,11]. As such, identifying the intrinsic effect of RSE-induced fatigue on mechanical alterations also requires pre-to-post assessments to be conducted at similar constant velocities.

When exercising under heat stress, there is compelling evidence to suggest that elevations in whole-body temperature increase cardiovascular strain [12,13] and alter neuromuscular function [14,15], thus hampering repeated-sprint exercise (RSE) performance (for review see [16]). Although the influence of heat stress on performance during RSE has been explored in cycling [17,18], there appears to be a paucity of data describing the alterations in running mechanics when sprinting repeatedly in a hot environment. Moreover, whether an accentuated thermal strain exerts a negative impact on stride kinematics and spring-mass parameters during constant-velocity running performed prior to and following multiple-set RSE remains to be determined.

Therefore, the aim of this study was to comprehensively investigate the effect of heat stress on i) fatigue-induced changes in performance and the associated alterations in running mechanics during multiple-set RSE and ii) on constant-velocity running mechanics and spring-mass behaviour after multiple-set RSE. This was accomplished by measuring tri-dimensional ground reaction forces with the use of a modified instrumented sprint treadmill. It was hypothesized that the development of thermal strain (*i*.*e*., elevated core and skin temperatures) under heat stress would exacerbate the magnitude of stride mechanical alterations (both during sprinting and running at constant velocity), due to the multiple-set nature of the RSE.

## Methods

### Participants

Fourteen male volunteers (mean±SD age, 31.3±4.5 years; stature, 176.2±4.7 cm; body weight, 74.5±8.5 kg) who were recreationally active (4.8±2.7 h.wk^-1^) in intermittent sports (*i*.*e*., football, futsal, tennis, squash) took part in the study. All participants were free of musculoskeletal pain or injuries. In the 6 months preceding the study, their training included activity-specific (*i*.*e*., technical and tactical skills), aerobic (*i*.*e*., continuous and intermittent) and anaerobic (*i*.*e*., strength, sprints, change of direction) exercise sessions. Participants were also asked to avoid strenuous exercise in the 48 h preceding their visits to the laboratory, as well as refrain from caffeine for 12 h and alcohol for 24 h. Although the study took place in Qatar, the participants were not accustomed to sprinting in the heat, as the study was conducted in the winter (mean ambient temperature of 20–25°C). Participants wore standardized personal athletic training attire (T-shirt, shorts, socks, and running shoes). Written informed consent was obtained from all participants. The study was approved by the *Shafallah Medical Genetics Center* Ethics committee (IRB Project Number 2011–011) and conducted according to the Declaration of Helsinki.

### Protocol overview

**Familiarization.** Approximately 1 week prior to testing, participants completed a pre-experimental session in temperate conditions including habituation runs of ~30 s at 10 and 20 km.h^-1^, followed by short (<5 s) familiarization treadmill sprints at increasing velocities with full recovery, until feeling comfortable with the running technique expected (this generally required 7–10 sprints). Then, they performed three maximal 5-s sprints, separated by 2 min of passive rest. All participants satisfied the criteria of having a coefficient of variation <2.2% for distance covered across three successive trials [5]. After 10 min of rest, they completed the RSE protocol in full (see *Experimental trials)*. Strong verbal encouragement was given during all maximal efforts.

**Experimental trials.** Participants performed a multiple-set RSE protocol composed of three sets of five 5-s sprints with 25 s of passive rest between sprints and 3 min between sets in HOT (37.6±2.3°C; 21.4±3.1% relative humidity) and CON (24.9±0.6°C; 45.3±7.8% relative humidity) conditions. The trials were conducted in a randomized order at the same time of day (±1h) and separated by at least 4 days. Upon arrival to the laboratory, participants were instrumented for physiological measurements (see *Responses to exercise*). Thereafter, participants performed a standardized ~30 min warm-up on the instrumented treadmill, which included 10 min of running (10 km.h^-1^), 15 min of athletic drills (skipping, high heels and butt-kicks), 3 × short burst accelerations (subjective “sense of effort” of 7, 8 and 9 over 10) [19], 2 × 3-s sprints (“sense of effort” of 8 and 9 over 10), and finally 3 × 5-s maximal sprints separated by 2 min of passive rest. The best of these three maximal sprints (greatest distance covered in 5 s) was used as the criterion score for the subsequent series to ascertain that no pacing occurred (See *Data analyses*). Afterwards, participants were required to perform 1-min runs at 10 km.h^-1^ then at 20 km.h^-1^ including an evaluation of stride mechanical patterns (see below). They were then allowed 5 min to rest in a standing position prior to undertaking the RSE protocol. Lastly, 3 min after the termination of RSE protocol, participants repeated the 10 and 20 km.h^-1^ runs whereby stride mechanical patterns were re-evaluated. The delay was necessary to allow sufficient recovery for successful completion of both the low and high velocity runs post-RSE, as well as a comparison to previous data [20]. Exposure to HOT conditions before commencement of the RSE protocol was ~40 min. The total duration of the testing session (*i*.*e*., from the beginning of the warm-up until the submaximal runs following the repeated-sprint ability test) was ~1 h and conducted in each respective environmental condition.

#### Instrumented sprint treadmill

All running was performed on an instrumented sprint treadmill (ADAL3D-WR, Medical Development–HEF Tecmachine, Andrézieux-Bouthéon, France). Briefly, it is mounted on a highly rigid metal frame fixed to the ground through four piezoelectric force transducers (KI 9077b; Kistler, Winterthur, Switzerland) and installed on a specially engineered concrete slab to ensure maximal rigidity of the supporting ground. This motorized treadmill allows participants to sprint due to the use of constant motor torque [3,5]. The motor torque, set to 160% of the default torque necessary to overcome the friction on the belt due to participant’s body weight [3], allows participants to sprint in a comfortable manner and produce their maximal effort without risking loss of balance.

A belt attached to a stiff rope (1 cm in diameter, ~2 m in length) was used to tether subjects to the 0.4-m vertical rail anchored to the wall behind them. An additional overhead safety harness with sufficient slack not to impede natural running mechanics was fastened to the participants to support them in the event of a fall. When correctly attached, participants could lean forward in a typical crouched sprint-start position with their left foot forward. This starting position was standardized and used in all sprint efforts. Following a 5-s verbal and visual countdown, the treadmill was released and the belt began to accelerate as participants applied a positive (*i*.*e*., propulsive) horizontal force.

#### Mechanical variables

Data were continuously sampled at 1000 Hz. After appropriate filtering (Butterworth-type 30 Hz low-pass filter; Adirun, Tecmachine, Andrézieux-Bouthéon, France), instantaneous vertical, net horizontal and total (*i*.*e*., resultant) ground reaction forces were averaged for each support phase (vertical force above 30 N) over the 5-s sprints and the 1-min runs at low (i.e., 10 km.h^-1^) and high (i.e., 20 km.h^-1^) constant velocities, and expressed in units of the body’s weight (BW). These data were completed by measurements of the main step kinematic variables: contact time (s), aerial time (s), swing time (s), step frequency (Hz) and step length (m). Lastly, for each 5-s sprint, horizontal forces were used with the corresponding average belt velocity to compute net power output in the horizontal direction (propulsive power = horizontal force × running velocity, W.kg^−1^).

A linear spring-mass model of running was used to investigate the main mechanical parameters characterizing the lower limb behavior during running [21,22]. Vertical stiffness (kN.m^-1^) was calculated as the ratio of peak vertical forces (N) to the maximal vertical downward displacement of center of mass (m), which was determined by double integration of vertical acceleration of center of mass over time during ground contact. Leg stiffness (kN.m^-1^) was calculated as the ratio of peak vertical forces to the maximum leg spring compression [maximal vertical downward displacement + L_0_ - √L_0_^2^ –(0.5 × running velocity × contact time)^2^, m], both occurring at mid-stance. Initial leg length (L_0_, great trochanter to ground distance in a standing position) was determined from participant’s stature as L_0_ = 0.53 × stature [23]. During the 1-min runs, vertical mean loading rate was calculated as the mean value of the time-derivate of vertical force signal within the first 50 ms of the support phase, and expressed in body weight.s^-1^.

#### Responses to exercise

**Hydration and temperature measurements.** Upon arrival on testing days, participants provided a urine sample for the measurement of urine specific gravity (Pal-10-S, Vitech Scientific Ltd. West Sussex, UK). A telemetric temperature pill (VitalSense®, Mini Mitter, Respironics, Herrsching, Germany) used to monitor core temperature was then inserted the length of a gloved index finger beyond the anal sphincter. Skin temperatures of the chest, upper arm, thigh and lower leg were monitored via temperature monitor/data loggers (iButtons, maxim integrated, USA) and used to calculate mean skin temperature with the weighted coefficient proposed by Ramanathan [24]: chest 30%, upper arm 30%, thigh 20%, and lower leg 20%. All temperatures were recorded at 1 min intervals. Participants were permitted to drink water (20–22°C) *ad libitum* during the trials. Body weight changes, corrected for fluid ingested and sweat trapped in clothing, were evaluated at the conclusion of each trial.

**Physiological and perceptual measurements.** Heart rate was monitored telemetrically with a Polar transmitter-receiver (T-31; Polar Electro, Lake Success, NY, USA) and recorded every 5 s. Thermal comfort [25] scores were recorded on a seven-point scale, and ratings of perceived exertion (RPE) on the Borg 6–20 scale [26] exactly 10 s after each sprint. A capillary blood sample was taken from the fingertip and analyzed for blood lactate concentration with the Lactate Pro (LT-1710, Arkray, Japan) portable analyzer at baseline, after warm-up and at end-exercise (*~*2 min post-RSE).

### Data analyses

To prevent pacing effects occurring during the RSE protocol, participants were required to achieve at least 95% of their criterion score (determined at the end of the warm-up procedure) during the first sprint of the multiple-set RSE for each testing session. The 95% criterion score was satisfied for 11 out of 14 participants in both conditions [mean±SD (range): 96.7±2.8% (96.7–101.9%) and 101.2±3.2% (96.5–108.4%) in HOT and CON, respectively]. The three participants that covered a distance below 95% of that measured during the reference sprints (*i*.*e*., criterion score range: 82.2–94.1%) were removed from our original participants sample (n = 14), which finally included a total of 11 participants. Mechanical data consisted of the continuous (step-by-step) measurement of running kinetics and kinematics for each sprint, which were averaged for each set for further analysis. Physiological and perceptual responses to exercise were also averaged across each set. During the 1-min constant-velocity runs, mechanical data for all steps collected over a 20-s sampling period (*i*.*e*., 38 to 58 s) were averaged for subsequent analysis [27].

### Statistical analysis

Two-way repeated-measures analysis of variance (ANOVA) [Time (Baseline, Set 1, Set 2 and Set 3 or Baseline, Pre-RSE and Post-RSE) × Condition (HOT and CON)] were used to compare running thermal, physiological and perceptual responses. Two-way repeated-measures analysis of variance (ANOVA) [Time (Set 1, Set 2 and Set 3) × Condition (HOT and CON)] were used to compare running performance and mechanical data during sprinting. For low and high constant-velocity running, separate two-way repeated-measures of variance [Time (Pre-RSE and Post-RSE) × Condition (HOT and CON)] were used to compare mechanical data. Mauchly’s tests of sphericity were performed to assess assumptions of variance, and a Greenhouse-Geisser correction was performed to adjust the degree of freedom when applicable. Bonferroni post-hoc multiple comparisons were performed in case of significant main effects. Partial eta-squared were calculated as a measure of effect size (indicated in the Tables were applicable), with values of 0.01, 0.06 and >0.14 considered as small, medium and large, respectively [28]. All statistical calculations were performed using SPSS statistical software V.21.0 (IBM Corp., Armonk, NY, USA). The significance level was set at P<0.05. Values are expressed as means±SD.

## Results

### Hydration and temperature responses

**Fig 1:**
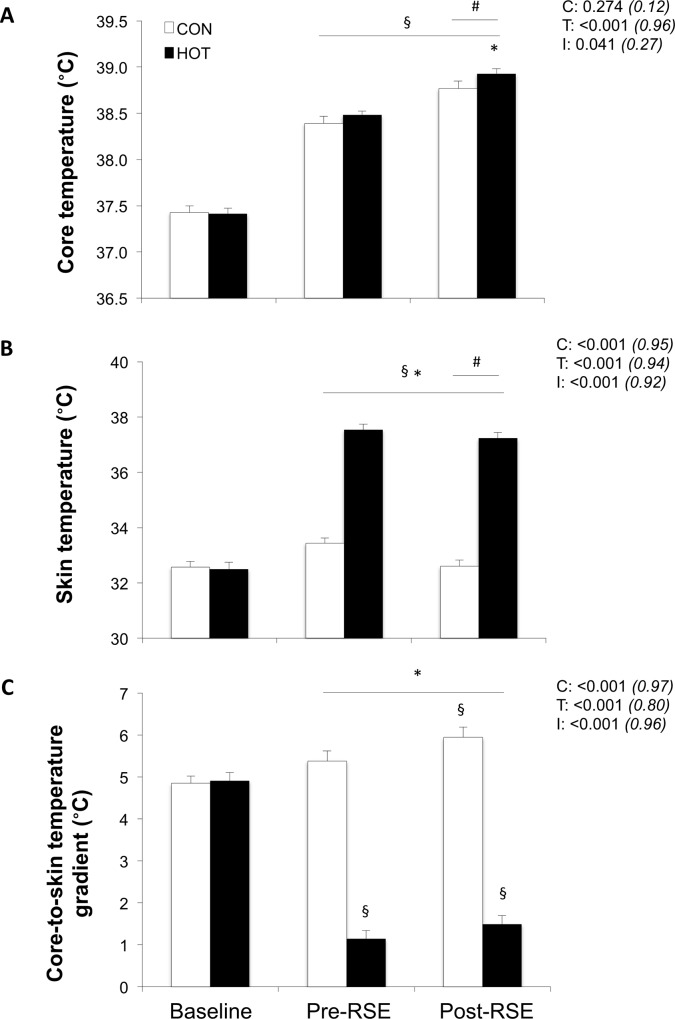
**Core temperature (A), skin temperature (B) and core-to-skin temperature gradient (C) at baseline, before (Pre-RSE) and after (Post-RSE) the repeated-sprint exercise in HOT and CON conditions.** Values are mean±SD (n = 11). C, T, and I respectively refer to ANOVA main effects of condition, time, and interaction between these two factors with P-value and partial eta-squared in parentheses. * significantly different from CON, P<0.05. ^§^ significantly different from Baseline, P<0.05. # significantly different from Pre-RSE, P<0.05.

Pre-exercise urine specific gravity was similar between HOT (1.012±0.009) and CON (1.015±0.009). A greater volume of water was consumed during HOT (795±399 ml.hr^-1^) *vs*. CON (418±252 ml.hr^-1^; P<0.001). From Pre- to Post-RSE, percent body weight loss was larger in HOT (-0.7±0.4%) *vs*. CON (-0.5±0.3%; P<0.05). Mean skin (37.2±0.7 *vs*. 32.7±0.8°C; P<0.001) and core (38.93±0.20 *vs*. 38.77±0.31°C; P<0.05) temperatures were higher and the core-to-skin temperature gradient narrower (1.49±0.76 *vs*. 5.94±0.91°C; P<0.001) following RSE in HOT than in CON (Fig 1).

### Physiological and perceptual responses

Heart rate, ratings of perceived exertion and thermal comfort data are presented in Table 1. Blood lactate concentration increases from baseline to Pre and Post-RSE were lower in HOT (1.5±0.3 to 5.9±2.2 and 11.4±2.9 mmol.L^-1^) compared to CON (1.7±0.4 to 7.2±2.5 and 13.3±2.7 mmol.L^-1^) (P<0.05).

**Table 1:** Physiological and perceptual responses for each of the three sets of the repeated-sprint exercise protocol in HOT and CON conditions.

Running kinematics	Condition	Baseline	Set 1	Set 2	Set 3	ANOVA *(η*^*2*^*)*		
						Condition	Time	Interaction
Heart rate (bpm)	HOT	68.0±11.5	162.7±10.1*^§^	160.9±9.8*^§^	162.9±10.4*^§^	**0.035**	**<0.001**	0.261
	CON	68.7±10.9	158.1±13.1^§^	157.7±11.2^§^	159.9±9.4^§^	*(0*.*37)*	*(0*.*99)*	*(0*.*13)*
Ratings of perceived exertion	HOT	-	15.6±1.8*	17.1±1.6*†	17.6±1.6*†	**0.003**	**0.001**	0.196
	CON	-	14.5±2.2	15.6±2.3†	16.3±2.3†	*(0*.*59)*	*(0*.*63)*	*(0*.*16)*
Thermal comfort	HOT	4.1±0.8	5.4±0.6*^§^	5.7±0.7*^§^	6.2±0.6*^§^†*#*	**<0.001**	**<0.001**	**<0.001**
	CON	4.1±0.4	4.3±0.5^§^	4.4±0.6^§^	4.7±0.6^§^*#*	*(0*.*85)*	*(0*.*79)*	*(0*.*63)*

Values are mean ± SD (n = 11). η^**2**^, partial eta-squared values.

* significantly different from CON, P<0.05

^§^ significantly different from Baseline, P<0.05

† significantly different from Set 1, P<0.05

# significantly different from Set 2, P<0.05.

### Sprint mechanical data

**Fig 2:**
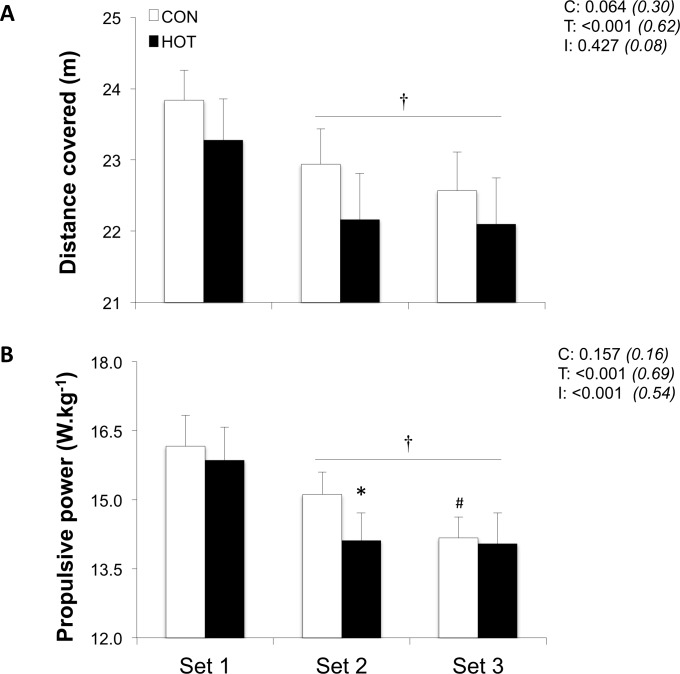
**Distance covered (A) and propulsive power (B) for each of the three sets of the repeated-sprint exercise in HOT and CON conditions.** Values are mean±SD (n = 11). C, T, and I respectively refer to ANOVA main effects of condition, time, and interaction between these two factors with P-value and partial eta-squared in parentheses. * significantly different from CON, P<0.05. † significantly different from Set 1, P<0.05. # significantly different from Set 2, P<0.05.

Distance covered (pooled condition values: -4.3±3.3% and -7.0±6.4%; P<0.001) decreased in sets 2 and 3 relative to set 1, with also a tendency for shorter distance covered (-2.7±3.4% for the average of sets 1–3) in HOT *vs*. CON (P = 0.06; Fig 2).

**Fig 3:**
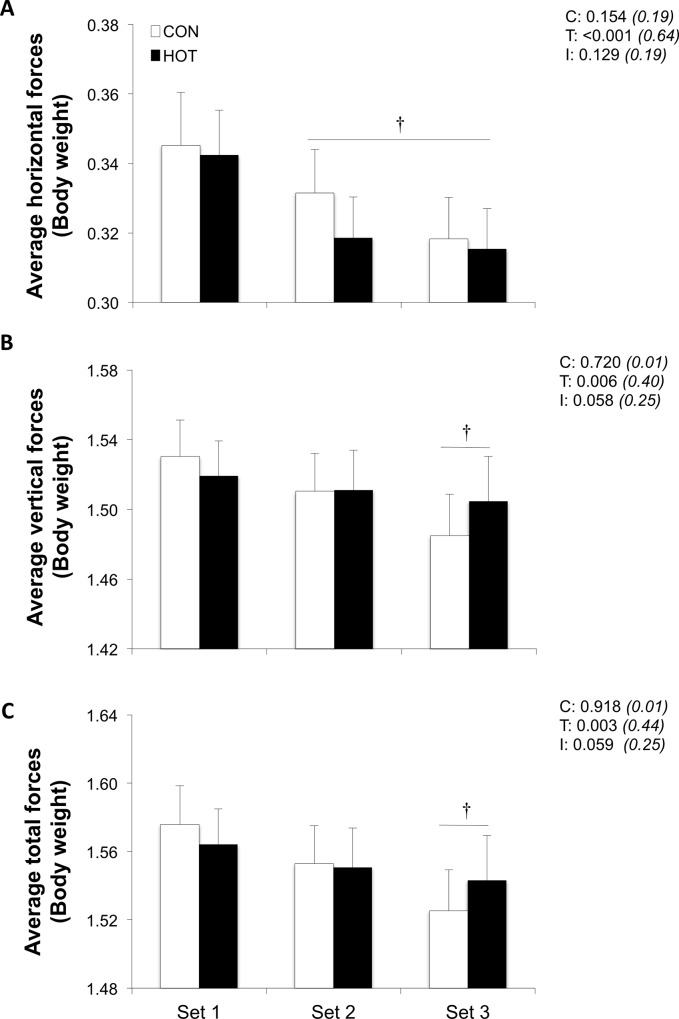
**Average horizontal (A), vertical (B) and total (C) ground reaction forces for each of the three sets of the repeated-sprint exercise in HOT and CON conditions.** Values are mean±SD (n = 11). C, T, and I respectively refer to ANOVA main effects of condition, time, and interaction between these two factors with P-value and partial eta-squared in parentheses. Forces are expressed in units of the body’s weight (BW). * significantly different from CON, P<0.05.

When pooling the two environmental conditions, average horizontal forces decreased by -5.2±1.2% and -9.1±3.0% in sets 2 and 3 respectively, relative to set 1 (both P<0.01; Fig 3). Average vertical and total forces also decreased from set 1 to set 3 (-2.6±5.5% and -3.0±2.8%, respectively, both P<0.05). Propulsive power (-6.0±1.2% and -16.9±2.4%; P<0.001) decreased in sets 2 and 3 relative to set 1 (Fig 2). There was a significant time × condition interaction for propulsive power (P<0.001): lower values were observed in set 2 in HOT *vs*. CON (-6.6%; P<0.05)

Contact time increased in sets 2 (+5.9±0.8%) and 3 (+9.6±1.6%) relative to set 1 (P<0.001), with higher mean values in HOT (+3.2±2.4%) across sets 1–3 (P<0.01) (Table 2). Step frequency decreased from set 1 to set 2 in both the HOT (4.8±3.8%; P<0.001) and CON (2.7±1.5%; P<0.01) conditions. Compared to CON, step frequencies were 2.6±1.6% (P<0.001) lower in HOT for the average of sets 1–3.

**Table 2:** Running kinematics for each of the three sets of the repeated-sprint exercise protocol in HOT and CON conditions.

Running kinematics	Condition	Set 1	Set 2	Set 3	ANOVA *(η*^*2*^*)*		
					Condition	Time	Interaction
Contact time (s)	HOT	0.165 ± 0.008	0.177 ± 0.010*†	0.179 ± 0.012*†	**0.002**	**<0.001**	0.241
	CON	0.163 ± 0.009	0.170 ± 0.010†	0.172 ± 0.011†	*(0*.*65)*	*(0*.*66)*	*(0*.*14)*
Aerial time (s)	HOT	0.086 ± 0.015	0.087 ± 0.014	0.087 ± 0.014	0.369	0.656	0.474
	CON	0.085 ± 0.013	0.084 ± 0.015	0.086 ± 0.016	*(0*.*08)*	*(0*.*66)*	*(0*.*47)*
Swing time (s)	HOT	0.335 ± 0.031	0.348 ± 0.029*†	0.350 ± 0.028*†	**0.007**	**0.001**	**0.046**
	CON	0.331 ± 0.027	0.337 ± 0.031†	0.341 ± 0.034†	*(0*.*53)*	*(0*.*63)*	*(0*.*26)*
Step frequency (Hz)	HOT	4.02 ± 0.28*	3.82 ± 0.26*†	3.79 ± 0.24*†	**<0.001**	**<0.001**	**0.042**
	CON	4.07 ± 0.28	3.96 ± 0.29†	3.92 ± 0.33†	*(0*.*73)*	*(0*.*71)*	*(0*.*27)*
Step length (m)	HOT	1.27 ± 0.20	1.28 ± 0.20	1.28 ± 0.20	0.456	0.821	0.302
	CON	1.27 ± 0.16	1.27 ± 0.17	1.26 ± 0.18	*(0*.*06)*	*(0*.*02)*	*(0*.*11)*

Values are mean ± SD (n = 11). η^**2**^, partial eta-squared values.

^*^ significantly different from CON, P<0.05.

† significantly different from Set 1, P<0.05.

Changes in spring-mass characteristics are displayed in Table 3. Independently of the condition, significant changes occurred from set 1 to set 3 in spring-mass characteristics with lower peak vertical forces (-2.5±3.5%), higher maximal center of mass vertical displacement (+12.9±10.5%) and leg compression (+6.5±0.6%), resulting in decreased vertical (-12.9±2.3%) and leg (-8.4±2.7%) stiffness values (all P<0.05). Maximal center of mass vertical displacement (-5.1±3.3%) and vertical stiffness (-5.5±5.5%) values for the average of sets 1–3 were lower in HOT *vs*. CON (P<0.001). No significant interaction effect was found for any spring-mass model parameter.

**Table 3:** Spring-mass model parameters for each of the three sets of the repeated-sprint exercise protocol in HOT and CON conditions.

Running kinematics	Condition	Set 1	Set 2	Set 3	ANOVA *(η*^*2*^*)*		
					Condition	Time	Interaction
Peak vertical forces (N)	HOT	1780 ± 250	1752 ± 251†	1747 ± 256†	0.705	**0.013**	0.730
	CON	1780 ± 236	1753 ± 248†	1759 ± 275†	*(0*.*02)*	*(0*.*35)*	*(0*.*03)*
Centre of mass vertical displacement (m)	HOT	0.031 ± 0.004*	0.034 ± 0.004*†	0.035 ± 0.004*†	**0.001**	**<0.001**	0.074
	CON	0.030 ± 0.004	0.032 ± 0.004†	0.033 ± 0.005†	*(0*.*69)*	*(0*.*65)*	*(0*.*23)*
Leg compression (m)	HOT	0.130 ± 0.023	0.136 ± 0.023	0.138 ± 0.023	0.157	**0.022**	0.188
	CON	0.129 ± 0.021	0.133 ± 0.022	0.132 ± 0.021	*(0*.*19)*	*(0*.*32)*	*(0*.*15)*
Vertical stiffness (kN.m^-1^)	HOT	58.3 ± 6.2*	52.2 ± 6.1*†	51.3 ± 6.3*†	**<0.001**	**<0.001**	0.134
	CON	59.8 ± 5.7	55.9 ± 6.0†	55.0 ± 7.9†	*(0*.*75)*	*(0*.*71)*	*(0*.*13)*
Leg stiffness (kN.m^-1^)	HOT	14.9 ± 3.4	13.8 ± 3.1†	13.4 ± 2.6†	0.379	**0.003**	0.251
	CON	14.9 ± 1.8	14.1 ± 2.1†	14.3 ± 2.7†	*(0*.*08)*	*(0*.*44)*	*(0*.*13)*

Values are mean ± SD (n = 11). η^**2**^, partial eta-squared values.

* significantly different from CON, P<0.05.

† significantly different from Set 1, P<0.05.

### Constant-velocity runs

Completion of the RSE did not induce any changes in constant-velocity running pattern, be it at low (10 km.h^-1^; Table 4) or high (20 km.h^-1^; Table 5) velocity, except for step frequency over time (P = 0.047, *η*^2^ = 0.34) and between conditions (P = 0.021, *η*^2^ = 0.43), as well as step length between conditions (P = 0.018, *η*^2^ = 45). No interaction effect was found for any mechanical parameter.

**Table 4:** Constant low (10 km.h^-1^) velocity running kinematics and spring-mass variables prior to (PRE-RSE) and after (POST-RSE) the multi-set repeated-sprint exercise (RSE) in HOT and CON conditions.

	PRE-RSE		POST-RSE		ANOVA (*η*^*2*^)		
	HOT	CON	HOT	CON	Condition	Time	Interaction
**Running kinematics**							
Contact time (s)	0.266±0.017	0.264±0.019	0.267±0.016	0.263±0.016	0.115	0.950	0.358
					*(0*.*23)*	*(0*.*01)*	*(0*.*09)*
Aerial time (s)	0.105±0.023	0.103±0.022	0.098±0.018	0.100±0.018	0.966	0.105	0.305
					*(0*.*01)*	*(0*.*24)*	*(0*.*11)*
Step frequency (Hz)	2.70±0.12	2.73±0.11	2.75±0.10	2.76±0.10	**0.021**	**0.047**	0.524
					*(0*.*43)*	*(0*.*34)*	*(0*.*52)*
Step length (m)	1.03±0.05	1.02±0.04	1.01±0.04	1.01±0.04	**0.018**	0.076	0.588
					*(0*.*45)*	*(0*.*28)*	*(0*.*03)*
**Spring-mass model parameters**							
Peak vertical forces (N)	1844±169	1832±176	1811±170	1816±192	0.687	0.261	0.352
					*(0*.*02)*	*(0*.*12)*	*(0*.*09)*
Centre of mass vertical displacement (m)	0.064±0.006	0.063±0.006	0.062±0.006	0.061±0.006	0.279	0.079	0.637
					*(0*.*12)*	*(0*.*28)*	*(0*.*02)*
Leg compression (m)	0.109±0.007	0.107±0.008	0.107±0.007	0.104±0.008	0.143	0.100	0.744
					*(0*.*20)*	*(0*.*25)*	*(0*.*01)*
Vertical stiffness (kN.m^-1^)	28.8±2.4	29.4±2.0	29.5±2.5	30.1±1.9	0.251	0.061	0.963
					*(0*.*13)*	*(0*.*30)*	*(0*.*01)*
Leg stiffness (kN.m^-1^)	17.0±1.4	17.2±1.3	17.0±1.3	17.5±1.4	0.142	0.507	0.489
					*(0*.*20)*	*(0*.*05)*	*(0*.*05)*
**Impact characteristics**							
Mean loading rate (BW.s^-1^)	44.8±10.5	44.4±9.8	44.1±9.2	44.7±9.5	0.913	0.864	0.433
					*(0*.*01)*	*(0*.*01)*	*(0*.*06)*

**Table 5:** Constant high (20 km.h^-1^) velocity running kinematics and spring-mass variables prior to (PRE-RSE) and after (POST-RSE) the multi-set repeated-sprint exercise (RSE) in HOT and CON conditions.

	PRE-RSE		POST-RSE		ANOVA (*η*^*2*^)		
	HOT	CON	HOT	CON	Condition	Time	Interaction
**Running kinematics**							
Contact time (s)	0.172±0.011	0.172±0.012	0.173±0.012	0.173±0.012	0.174	0.240	0.683
					*(0*.*18)*	*(0*.*14)*	*(0*.*02)*
Aerial time (s)	0.146±0.020	0.145±0.020	0.143±0.018	0.142±0.014	0.477	0.181	0.949
					*(0*.*05)*	*(0*.*17)*	*(0*.*01)*
Step frequency (Hz)	3.15±0.17	3.17±0.17	3.17±0.16	3.18±0.12	0.364	0.390	0.836
					*(0*.*08)*	*(0*.*08)*	*(0*.*01)*
Step length (m)	1.77±0.09	1.76±0.09	1.76±0.08	1.75±0.06	0.287	0.357	0.916
					*(0*.*11)*	*(0*.*09)*	*(0*.*01)*
**Spring-mass model parameters**							
Peak vertical forces (N)	2269±199	2261±202	2240±201	2225±198	0.365	0.078	0.785
					*(0*.*08)*	*(0*.*28)*	*(0*.*01)*
Centre of mass vertical displacement (m)	0.043±0.003	0.043±0.004	0.043±0.003	0.043±0.004	0.519	0.326	0.480
					*(0*.*04)*	*(0*.*10)*	*(0*.*05)*
Leg compression (m)	0.131±0.011	0.130±0.013	0.131±0.015	0.131±0.014	0.355	0.568	0.506
					*(0*.*09)*	*(0*.*03)*	*(0*.*05)*
Vertical stiffness (kN.m^-1^)	52.8±6.2	53.4±6.7	53.1±5.5	52.6±5.0	0.815	0.729	0.124
					*(0*.*01)*	*(0*.*01)*	*(0*.*22)*
Leg stiffness (kN.m^-1^)	17.4±1.5	17.5±1.6	17.2±1.9	17.1±1.8	0.678	0.215	0.308
					*(0*.*02)*	*(0*.*15)*	*(0*.*10)*
**Impact characteristics**							
Mean loading rate (BW.s^-1^)	102.1±21.2	100.9±21.6	103.4±17.3	99.1±18.2	0.108	0.910	0.299
					*(0*.*24)*	*(0*.*01)*	*(0*.*11)*

## Discussion

Although there is compelling evidence to suggest that compared to temperate conditions, larger performance decrements occur when repeated sprints are undertaken in hot environments due to the development of hyperthermia [16], the mechanical causes underlying these decrements are not completely understood. In the current study, the nature and the extent of fatigue-induced alterations in running mechanics during multiple-set RSE performed with and without environmental heat stress were examined for the first time. The major findings are that greater thermal (*i*.*e*., core and skin temperature) and perceptual strain during RSE under heat stress is associated with decrements in propulsive power, step frequency and vertical stiffness, along with longer contact time; however, running mechanical alterations induced by sprint repetitions were in general not more pronounced in hot compared with cooler ambient conditions. Furthermore, low and high constant-velocity running patterns displayed no pre-to-post RSE changes in either condition.

### Performance outcomes, physiological and perceptual responses

Compared to temperate environmental test settings, the detrimental effects of ambient temperatures >30°C on repeated-sprint ability are solely observed when exercise induces marked hyperthermia with core temperature exceeding 39°C [16]. Indeed, in hot (40°C) *vs*. temperate (20°C) conditions, the ability to produce power during five maximal 15-s sprints (rest = 15 s) was impaired when core (39.5°C) and muscle (40.2°C) temperatures were elevated following the completion of a 40 min submaximal intermittent sprint protocol in the heat [17]. The authors also noted that the added thermal strain imposed by the environment exacerbated the heart rate and RPE responses. In the present study (*i*.*e*., running mode and core temperature below 39°C), performance decreased as fatigue developed, with systematically lower distances covered, along with lower propulsive power values in HOT (mainly during set 2) (Fig 2). It was further observed that RSE was associated with elevated cardiovascular load (*i*.*e*., heart rate) and RPE in HOT *vs*. CON (Table 1). For repeated cycling sprints performed under heat stress (24 *vs*. 35°C) with a modest elevation in core temperature of ~0.5°C, participants experienced similar increases in heart rate and RPE, and were able to overcome the thermal sensations linked to the hot environment and to improve repeated-sprint ability [18]. In our study, greater levels of thermal strain, along with elevated cardiovascular and perceptual loads, accompanied the decrement in indices of repeated-sprint performance in the heat.

Another interesting observation is that most of the sprint mechanical alterations occurred between the first two sets, and notably when participants were exposed to heat stress. As a consequence of the narrow core-to-skin temperature gradient occurring in the HOT condition, it is likely that skin blood flow increased in order to meet the added requirement for heat dissipation [12]. Interestingly however, thermal comfort did not differ between set 1 and set 2 in either condition, with a larger heat-induced increase in thermal strain only observed during set 3. Contrastingly, completion of multiple-set RSE in temperate conditions has been associated with performance decrements during set 3 (but not 2) in reference to set 1 [9]. Because the nature of RSE (*i*.*e*., sprint duration, type of recovery, number of sprint repetitions), training status of participants and environmental conditions in which it is undertaken vary between studies, comparisons can only remain speculative. In line with the current findings however, was the observation of Serpiello et al. [9] that the reduction from set 1 to set 3 was two-to-three fold larger in power *vs*. velocity indices. In examining intra-session and inter-session reliability, it was recently recommended that the preferred indices to readily detect the smallest worthwhile changes in treadmill sprint performance should be the distance covered and the propulsive power [9]. This, along with the fact that only propulsive power displayed an interaction effect, reinforces the notion that mechanical parameters reflecting acceleration are the preferred variables to assess treadmill sprint performance.

### Sprint mechanical data

Given the short duration of the majority of sprints (<5 s) during team-sport competitions, a key physical attribute of many of these athletes is the ability to repeatedly accelerate to high velocities [29]. Although vertical force production has been linked to the ability to achieve high maximal running velocities in humans (*e*.*g*., [30]), horizontal forces and the associated forward orientation of resultant ground reaction force vectors have recently been put forward as major determinants of acceleration during running. For example, i) increasing running velocity from moderate (~40% of maximal running velocity) to “all-out” sprinting is more dependent on increments in horizontal than on vertical force production [11,31]; ii) at 8 m [32] and 16 m [33] from the start, applying ground reaction impulse in a more horizontal direction explains 44% and 61% of the variance of running velocity, respectively; and iii) the effectiveness of total forward direction force application greatly accounts for the difference in 40-m sprint performance between highly trained athletes [4]. During run-based RSE, reductions in the production of horizontal forces across successive efforts generally exceed those in the vertical direction [7,8], with larger fatigue levels (*i*.*e*., severe hypoxia compared to moderate hypoxia or normoxia) exacerbating the magnitude of these mechanical alterations [6]. Data from the current study confirm these observations with a three time greater reduction in horizontal forces relative to vertical or total forces from set 1 to set 3 (Fig 3). Moreover, we extend these observations to show that progressively less effective acceleration (*i*.*e*., horizontal forces) across sets were not exacerbated by heat stress.

As with previous RSE studies [6–8], this study showed that step frequency changes more dramatically as participants fatigue than does step length. With larger decreases in step frequency in HOT *vs*. CON during sets 2 and 3, these data reinforce that maintaining a faster step rate rather than taking longer steps is a prerequisite for optimizing performance when sprinting repeatedly in the heat. Additionally, an increase in contact time was observed with fatigue, but no alteration in aerial time (Table 2). This increase in ground contact time as sprints are repeated may be due, at least in part, to a reduced capacity of the neuromuscular system to generate force rapidly (*i*.*e*., impaired stretch-shortening cycle efficiency) [34]. In support of this, decrements in rapid force development (*i*.*e*., within 100–200 ms of contraction onset) in the knee extensors have recently been observed after the completion of eight treadmill sprints [35]. Playing tennis, which involves numerous taxing lower extremity stretch-shortening cycles (*i*.*e*., directional changes), results in similar fatigue-related alterations in explosive strength in hot and temperate environments [36].

The classic linear spring-mass model, derived from vertical force-time waveforms, is increasingly used to explain many aspects of running gaits with remarkable accuracy given its mechanical simplicity [23]. In this model, the single-mass approach models running individuals as a lumped point-mass mass bouncing on a massless leg spring [21]. As highlighted in the Table 3, a decrease in peak vertical forces occurred during multiple-set RSE in HOT and CON conditions, along with increases in maximal vertical displacement and leg compression, causing reductions in vertical and leg stiffness values. These data are supported by recent investigations examining the effect of RSE-induced fatigue on changes in spring-mass characteristics [6,7]. The current results however, are novel in showing that globally that maximal vertical displacement and vertical stiffness were negatively affected when completing the RSE in HOT *vs*. CON. Collectively, the spring-mass results confirm that mechanical behavior changes towards a lower stride mechanical efficiency were exacerbated with exacerbated heat stress. Notwithstanding, in the absence of an interaction effect for any spring-mass model parameter, it must be acknowledged that reductions in the rate of force transmission between the legs and the ground are comparable between the two environments. Future studies should specifically analyze the respective contributions of ankle, knee and hip joint angles in relation to these more general adjustments of the leg spring.

### Constant-velocity runs

The current findings display no pre-to-post RSE changes in low and high constant-velocity running patterns in both conditions (Tables 4 and 5). These data corroborate previous observations made using the ADAL treadmill showing a similar range of values for mechanical properties at the same running velocities (*i*.*e*., 10 and 20 km.h^-1^) following the completion of four sets of five 6-s sprints with 24-s recovery and 3 min between sets in temperate conditions [20]. Similarly unchanged stride kinematics as well as leg and vertical stiffness values were observed after an exhaustive 2000-m run on an indoor track at constant velocity (13 km.h^-1^) [37]. Taken as a whole, the absence of changes in constant-velocity running patterns suggests a strong link between fatigue-induced velocity decrements during sprinting and mechanical alterations.

Although it is not yet clear which impact loading variable has the most important association with running-related injury risk [38], results from a recent meta-analysis have highlighted that reducing the loading rate of the vertical ground reaction force (*e*.*g*., via increasing frequency [39] and/or forefoot striking [40]) by 10–15% may help in preventing stress fractures occurrence [41]. Unique to this study, however, was a lack of change in loading rate values during constant-velocity runs throughout the protocol in either condition (Tables 4 and 5). These data suggest that multiple-set RSE conducted under heat stress does not further modify the ability of athletes to cushion impact during constant-velocity running. In contrast, although the first effort of six 30-s runs at 115% of the velocity associated with maximal oxygen uptake (*i*.*e*., 19.9±0.7 km.h^-1^) with 30-s passive recovery appears in line with the present high constant-velocity running patterns, the mean loading rates have been shown to increase by ~7% from the first to the last repetition [27]. Extending the timeframe during which constant-velocity running is evaluated may allow for identifying whether RSE influences running mechanics and spring-mass characteristics.

### Additional considerations / limitations

In the present study, a possible reason for the lack of a larger difference in running mechanical performance between the two environments could be the ~0.15°C difference in core temperature after set 3, with participants reaching a core temperature >38.5°C in CON (Fig 1). The attainment of this core temperature in CON may partly be explained by the completion of a vigorous 30-min warm-up, which increased core temperature by ~1°C in both conditions. In the RSE-related literature, brief warm-up procedures (<10 min with 1–3 “all-out” efforts) are conducted (*e*.*g*., [9]), calling into question the ability of participants to truly perform maximal efforts from the initial sprint. Additionally, the fact that relative humidity was ~24% higher in the CON condition may have reduced evaporative heat loss compared to the HOT environment. Indeed, humid environments compromise evaporative heat loss and decrease exercise tolerance [42]. Future studies are thus required to examine the effect of adjusting levels of humidity to elicit similar absolute skin-air vapor pressure gradients at different ambient air temperatures to produce a similar drive for evaporation on repeated-sprint ability.

Certain limitations must be acknowledged. Firstly, the participants were recreational team-sport players and therefore do not necessarily mirror a cohort of elite competitors, which implies that conclusions must remain specific to the population tested. Secondly, to reflect true or ecological running mechanics, participants should perform over-ground sprints with their foot strikes recorded by a number of force plates laid in series. However, given the relationship between over-ground and treadmill sprint mechanics [4], it would seem that the latter provides a practical alternative and a reliable measure of stride mechanical efficiency whilst sprinting [5].

### Practical implications

- Defining the biomechanical adjustments that occur under situations of environmental heat stress, may represent a useful approach for tailoring training routines with the goal of improving repeated-sprint ability. This could be of interest for outdoor sports (*e*.*g*., football) played in Equatorial or Middle-Eastern regions, where maximal accelerations are repeated over the course of games played in the heat.
- Sub-maximal constant-velocity running pattern assessments may be used when studying the mechanical alterations related to repeated sprinting in order to distinguish the fatigue-related changes from those related to reductions in running velocity.”

## Conclusion

In summary, higher thermal and perceptual strain during multiple-set RSE under heat stress is associated with decrements in propulsive power, step frequency and vertical stiffness, along with longer contact time. However, running mechanical alterations induced by sprint repetitions were in general not more pronounced in hot compared with cooler ambient conditions. Moreover, completion of multiple-set RSE did not influence 1-min low and high constant-velocity (*i*.*e*., 10 and 20 km.h^-1^) running patterns. The absence of changes in constant-velocity running patterns therefore suggests a strong link between fatigue-induced velocity decrements during sprinting and mechanical alterations. While these results shed light on the biomechanical manifestation of fatigue when athletes repeatedly sprint at their maximum under heat stress, additional studies are needed to demonstrate how to improve RSE tolerance and optimize performance when competing in a hot environment. Given the preponderant role of cardiovascular and perceptual strain on performance and mechanical alterations, future studies should consider RSE inducing heavier thermal strain and the impact of potential countermeasures (*e*.*g*., pre-cooling, acclimation) aimed at mitigating its influence.
